# Targeted gene silencing using a follicle-stimulating hormone peptide-conjugated nanoparticle system improves its specificity and efficacy in ovarian clear cell carcinoma in vitro

**DOI:** 10.1186/1757-2215-6-80

**Published:** 2013-11-20

**Authors:** Shanshan Hong, Xiaoyan Zhang, Jun Chen, Jiabing Zhou, Yufang Zheng, Congjian Xu

**Affiliations:** 1 Obstetrics and Gynecology Hospital, Fudan University, Shanghai 200011, People’s Republic of China; 2 Department of Obstetrics and Gynecology of Shanghai Medical School, Fudan University, Shanghai 200032, People’s Republic of China; 3 Shanghai Key Laboratory of Female Reproductive Endocrine Related Diseases, Shanghai 200011, People’s Republic of China; 4 Department of Pharmaceutics, School of Pharmacy, Fudan University, Shanghai 201203, People’s Republic of China; 5 School of Life Sciences, Fudan University, Shanghai 200433, People’s Republic of China; 6 Institute of Biomedical Sciences, Fudan University, Shanghai 200032, People’s Republic of China

**Keywords:** Ovarian carcinoma, Targeted therapy, Follicle-stimulating hormone, Growth-regulated oncogene α, Short interfering RNA, Nanoparticle

## Abstract

**Background:**

RNA interference technology has shown high therapeutic potential for cancer treatment. However, serum instability, poor tissue permeability and non-specific uptake of short interfering RNA (siRNA) limit its administration *in vivo*. To overcome these limitations and improve the specificity for ovarian cancer, we developed a targeted nanoparticle delivery system for siRNA. This system included follicle-stimulating hormone (FSH) β 33–53 peptide as a targeting moiety that specifically recognized FSH receptor (FSHR) expressed on ovarian cancer cells. Growth regulated oncogene α (gro-α) has been reported to be involved in ovarian cancer development and progression. Thus, siRNA targeted to gro-α was used as an antitumor drug in this delivery system.

**Methods:**

FSH β 33–53 peptide-conjugated gro-α siRNA-loaded polyethylene glycol (PEG)-polyethylenimine (PEI) nanoparticles (FSH33-G-NP) were prepared and characterized by gel retardation assay and transmission electron microscopy. Particle size and zeta potential were determined. Expression of gro-α mRNA and protein was detected by real-time quantitative RT-PCR, immunocytochemistry and enzyme-linked immunosorbent assay. The proliferation, migration and invasion of the ovarian clear cell carcinoma cell line ES-2 were evaluated by cell counting kit-8 assay, cell scratch assay and transwell migration assay.

**Results:**

A siRNA sequence that is effective in silencing gro-α expression was obtained and loaded into the targeted delivery system. Compared with gro-α siRNA-loaded nanoparticles without FSH peptide modification (G-NP), FSH33-G-NP significantly down-regulated gro-α expression in ES-2 cells at mRNA and protein levels. Consequently, the aggressive biological behaviors of ES-2 cells, including proliferation, migration and invasion, were suppressed after silencing gro-α expression, and the addition of the FSH β 33–53 peptide enhanced the suppressive effects.

**Conclusions:**

This study indicated that a FSHR-mediated delivery system could mediate the highly selective delivery of siRNA into ovarian cancer cells and that silencing gro-α expression could be a potential choice for ovarian cancer treatment.

## Background

Ovarian cancer, known as a “silent killer”, is the most fatal of all female reproductive system malignant tumors due to the lack of effective early diagnostic methods and because most late-stage patients do not respond to routine treatments [1]. When compared with other epithelial ovarian cancers, ovarian clear cell carcinoma is more easily resistant to conventional platinum-based chemotherapy and has a worse prognosis [2]. Therefore, more effective treatment methods are urgently needed.

Short interfering RNA (siRNA) has been considered as a useful tool to silence genes. Recently, RNA interference (RNAi) technology has shown high therapeutic potential for cancer treatment [3]. However, siRNA administration *in vivo* is limited because of its serum instability, poor cellular membrane permeability and non-specific uptake [4]. Delivery systems including liposomes, nanoparticles (NPs), chemical modification and viral vectors have been used to overcome these limitations [5,6]. To improve the specificity and selectivity for tumor tissue, specific ligands including monoclonal antibodies, peptides and small molecules have often been introduced into the delivery systems [7].

In our previous study, we showed that follicle-stimulating hormone (FSH) β 33–53 peptide and FSH β 81–95 peptide are able to facilitate the access of paclitaxel-loaded NPs specifically into FSH receptor (FSHR)-positive ovarian tissues. In addition, paclitaxel NPs modified with FSH β 33–53 peptide or FSH β 81–95 peptide showed a higher antitumor efficacy against ovarian cancer and produced fewer adverse side effects [8,9]. FSHR-mediated targeted therapeutics show high potential in ovarian cancer therapy because of limited FSHR distribution in the human reproductive system. To specifically deliver genetic drugs including siRNA into ovarian cancer tissues, we recently developed a novel gene delivery system, polyethylene glycol (PEG)-polyethylenimine (PEI) complex modified with FSH β 33–53 peptide, to deliver siRNA carried by NPs into FSHR-positive cells [10].

Growth regulated oncogene α (gro-α), also called chemokine (C-X-C motif) ligand 1, is secreted by macrophage, neutrophil and epithelial cells, and gro-α plays a role in angiogenesis, inflammation and wound healing [11]. There is high level of gro-α expression in ulcerative colitis, colon adenomas, colon cancer, melanoma, breast cancer, bladder cancer and ovarian cancer [12-17]. Gro-α overexpression could promote the proliferation, invasion and metastasis of tumor cells [18,19]. Recent studies have shown that tissues and sera from patients with ovarian cancer have high levels of gro-α expression, while normal ovarian epithelial cells and fibroblasts have lower gro-α expression [20]. High levels of gro-α in stromal cells promote the senescence of fibroblasts and consequently cause the malignant transformation of ovarian epithelial cells [20,21]. Moreover, gro-α over expression can promote the development and progression of ovarian cancer and the formation of endometriosis [22]. Thus, the down-regulation of gro-α might suppress the aggressive biological behaviors of ovarian cancer cells.

In this study, to overcome the limitations of siRNA administration *in vivo* and improve the specificity for ovarian cancer, we prepared FSH β 33–53 peptide-conjugated gro-α siRNA-loaded nanoparticle. FSH β 33–53 peptide was used as an ovarian cancer targeting moiety, and siRNA targeted to gro-α was used as a therapeutic drug. The specific down-regulation of gro-α and the suppression of aggressive biological behaviors of ovarian clear cell carcinoma cells were further evaluated after treatment.

## Methods

### Materials

FSH β 33–53 peptide (YTRDLVYKDPARPKIQKTCTF) was synthesized by China Peptides Co., Ltd. (Shanghai, China). Branched PEI (MW 25,000 Da) was purchased from Sigma Aldrich Co. (St. Louis, USA). Maleimide-conjugated PEG (Mal-PEG) was purchased from Nektar Therapeutics (San Carlos, CA). The siSTABLE siRNA sequences targeted to gro-α mRNA and negative control siRNA (siRNA-NC) were synthesized by Thermo Fisher Scientific (Shanghai, China). The sequences were as follows: 5*′*-GCUGGCGGAUCCAAGCAAA-3*′* (siRNA-1), 5*′*-TTTCCGCCCATTCTTGAGTGT-3*′* (siRNA-2), 5*′*-CAAGCTTTCCGCCCATTCTTG-3*′* (siRNA-3) and 5*′*-TATAATAGGACAGTGTGCAGG-3*′* (siRNA-4). The siRNA expression plasmid, pcDNA™6.2-GW/EmGFP-miR (5,699 bp), was obtained from Invitrogen Trading Co., Ltd. (Shanghai, China). DharmaFECT transfection reagent was obtained from Thermo Fisher Scientific (Shanghai, China). FSHR antibody and gro-α antibody were purchased from Abcam Ltd. (San Francisco, USA). The gro-α ELISA kit was purchased from R&D Systems Inc. (Minneapolis, USA). The cDNA synthesis kit was purchased from Fermentas Inc. (Canada). The Cell Counting Kit-8 (CCK-8) was purchased from Dojindo Laboratories (Kumamoto, Japan).

### Cell culture

The human serous ovarian carcinoma cell line SKOV-3 and human ovarian clear cell carcinoma cell line ES-2 were purchased from the Cell Bank of the Chinese Academy of Science (Shanghai, China). SKOV-3 cells were grown in McCoy”s 5A Medium, and ES-2 cells were grown in RPMI 1640 medium. Medium was supplemented with 10% fetal bovine serum, and cells were cultured at 37°C in a 5% CO_2_ environment.

To screen for an effective siRNA sequence targeting gro-α, ES-2 cells were seeded in 24-well plates at a density of 1 × 10^5^ cells per well and cultured to reach 60% confluence. Then, 1.5 μg of siRNA-1, siRNA-2, siRNA-3, siRNA-4 or siRNA-NC along with DharmaFECT transfection reagent were diluted and added to the corresponding wells according to the manufacturer’s instructions. After incubation for 4 h, the medium containing siRNA was replaced with fresh medium containing 10% fetal bovine serum. After 24 h or 48 h, the cell lysates were collected for reverse transcription-polymerase chain reaction (RT-PCR), and cell supernatants were collected for enzyme-linked immunosorbent assay (ELISA).

To detect the suppression efficiency of gro-α by nanoparticle complexes, the same procedures were performed as above, except that the cells were incubated with serum-free medium containing 1.5 μg of gro-α siRNA-loaded nanoparticles without the transfection reagents.

### Preparation and characterization of FSH β 33–53 peptide-conjugated gro-α siRNA-loaded NPs

The gro-α siRNA4-loaded nanoparticle complexes with or without FSH β 33–53 peptide modification were prepared as previously described [10]. Briefly, the solutions containing FSH β 33–53 peptide and Mal-PEG were mixed and magnetically stirred for 6 h at room temperature. Then, 46.5 mg of this product, FSH33-PEG, was added to 10 mL of 2 mg/mL PEI solution and magnetically stirred for 24 h at room temperature. The product, FSH33-PEG-PEI, was dissolved and added into the same volume of plasmid DNA (pDNA) (containing gro-α siRNA4) solution drop by drop with magnetically stirring. The molar ratio of nitrogen from PEI to phosphate from pDNA (N/P) was 25. The final complex, FSH33-PEG-PEI-pDNA, was freeze-dried. The PEG-PEI-pDNA complex without FSH peptide was prepared by the same method.

The encapsulating efficiencies of the complexes were determined by gel retardation assay. The morphologies of the complexes were examined using the Joel Jem 2100 F transmission electron microscope (JEOL Ltd., Japan). Particle size and zeta potential were determined using the Malvern Zetasizer autosize 4700 (Malvern Instruments Ltd., Malvern, UK).

### Immunocytochemistry

To detect the expression of FSHR and gro-α, immunocytochemistry was used. After fixation with 4% paraformaldehyde, cells were incubated with FSHR antibody (1:500) or gro-α antibody (1:500) overnight, and then incubated with peroxidase-conjugated anti-rabbit IgG for 30 min. The staining reaction was performed with diaminobenzidine. The cells were counter-stained with hematoxylin to detect nuclei, and imaged by light microscopy (Olympus, Tokyo, Japan).

### Reverse transcription-polymerase chain reaction

RNA was isolated from cells, and 1 μg of total RNA was reverse-transcribed using a cDNA synthesis kit according to the manufacturer’s instructions. The primers for detecting the gro-α gene were 5*′*-AAGAATGGGCGGAAAGC-3*′* (forward) and 5*′*-CTCCTAAGCGATGCTCAAAC-3*′* (reverse). The primers for the β-actin gene (ACTB) were 5*′*-TCCTTCCTGGGCATGGAGT-3*′* (forward) and 5*′*-CAGGAGGAGCAATGATCTTGAT-3*′* (reverse). PCR was performed as follows: 35 cycles consisting of denaturing at 95°C for 30 s, annealing at 55°C for 30 s, and extension at 72°C for 30 s with an initial denaturation of 5 min at 95°C and final extension of 5 min at 72°C. The PCR products were separated on a 1% agarose gel and analyzed using a UV imaging system.

For real-time quantitative RT-PCR (qRT-PCR), gro-α mRNA levels were determined with SYBR Green detection in a two-step reaction using the Eppendorf Mastercycler ep Realplex RT-PCR system (Eppendorf, Hamburg, Germany) as described previously [10]. Relative expression levels were calculated using the 2^-ΔΔCT^ method and were normalized to untreated control.

### Enzyme-linked immunosorbent assay

To investigate the gro-α levels secreted by cells, cell supernatants were collected after treatment and examined using a gro-α ELISA kit according to the manufacturer’s instructions. Briefly, samples and standards were added to ELISA microplates and incubated for 1.5 h at room temperature. After washing, samples were incubated with gro-α conjugate for 1 h at 4°C, and then with substrate solution for 15 min at room temperature. The reaction was stopped using stop solution. The optical density (OD) of final products was measured using a microplate reader (Thermo Fisher Scientific, Shanghai, China) at 450 nm wavelength.

### Cell proliferation analysis

Cells were seeded into 96-well plates at a density of 1 × 10^4^ cells per well and incubated overnight. Cells were incubated with serum-free medium containing 1.5 μg of gro-α siRNA-NP or FSH33-gro-α siRNA-NP for 4 h. Medium was then replaced with fresh medium containing 10% fetal bovine serum. At 24 h, 48 h, 72 h and 96 h, 10 μl of CCK-8 solution was added and the cells incubated for 1 h. The OD values were measured using a microplate reader at 450 nm wavelength. The inhibition rate was calculated relative to untreated cells.

### Cell migration assay

To study the effects of gro-α siRNA-loaded NPs on cell migration, a cell scratch assay was used. Cells in 24-well plates were treated as described above. After 72 h, the confluent cell monolayer was scraped with a 10 μl pipette tip. The cells were washed twice with medium and then cultured with serum-free medium. The cells were examined and photographed under light microscopy at 12 h and 36 h after scraping. The distances between one side of a scratch and the other were measured to evaluate cell migration ability.

### Cell invasion assay

A transwell migration assay was used to determine the effects of gro-α siRNA-loaded NPs on cell invasion. Cells in 24-well plates were treated as described above. After 24 h, cells were harvested and seeded into the upper chambers of transwell plates pre-coated with matrigel at a density of 1 × 10^4^ cells per well. After incubation for 24 h, cells were fixed by submerging the chambers in 4% paraformaldehyde for 30 min, and then stained with hematoxylin for 15 min. A cell count of migrated cells was determined by examining the chambers under light microscopy.

### Statistical analysis

Statistical analyses were performed using Student’s *t* test by SPSS software. The data were expressed as the mean ± SD, and a *P* < 0.05 was considered significant.

## Results

### Expression of FSHR and gro-α

**Figure 1:**
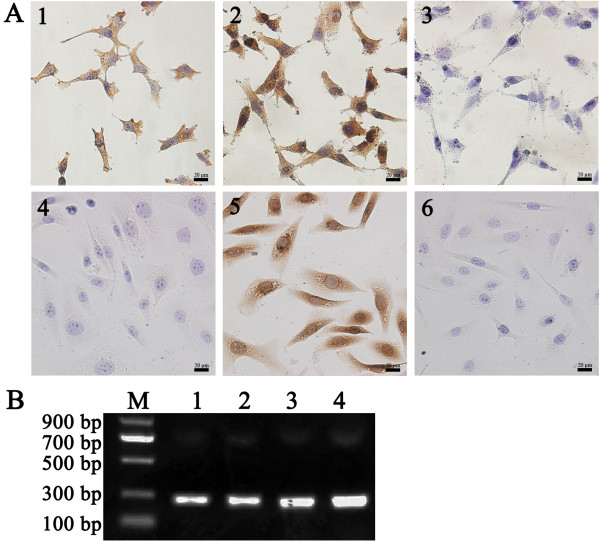
**Expression of FSHR and gro-α in human ovarian cancer cell lines SKOV-3 and ES-2. (A)** Detection of FSHR and gro-α expression by immunocytochemistry. 1, 2 and 3, ES-2 cells stained with FSHR antibody, gro-α antibody and control IgG, respectively; 4, 5 and 6, SKOV-3 cells stained with FSHR antibody, gro-α antibody and control IgG, respectively. Bar, 20 μm. **(B)** Detection of gro-α mRNA expression by RT-PCR. M, DNA marker; lanes 1 and 2, gro-α mRNA expression in ES-2 and SKOV-3 cells, respectively (226 bp); lanes 3 and 4, ACTB expression in ES-2 and SKOV-3 cells, respectively (208 bp).

To evaluate the possibility of using FSHR and gro-α as therapeutic targets, we examined FSHR and gro-α expression in two human ovarian cancer cell lines. ES-2 cells expressed FSHR, whereas SKOV-3 cells showed negative expression. Both cell lines expressed gro-α at protein and mRNA levels (Figure 1). To study targeted therapeutics in ovarian clear cell carcinoma, the human ovarian clear cell carcinoma cell line ES-2, which expressed both FSHR and gro-α, was used in this study.

### Screening of siRNA sequences targeted to gro-α

**Figure 2:**
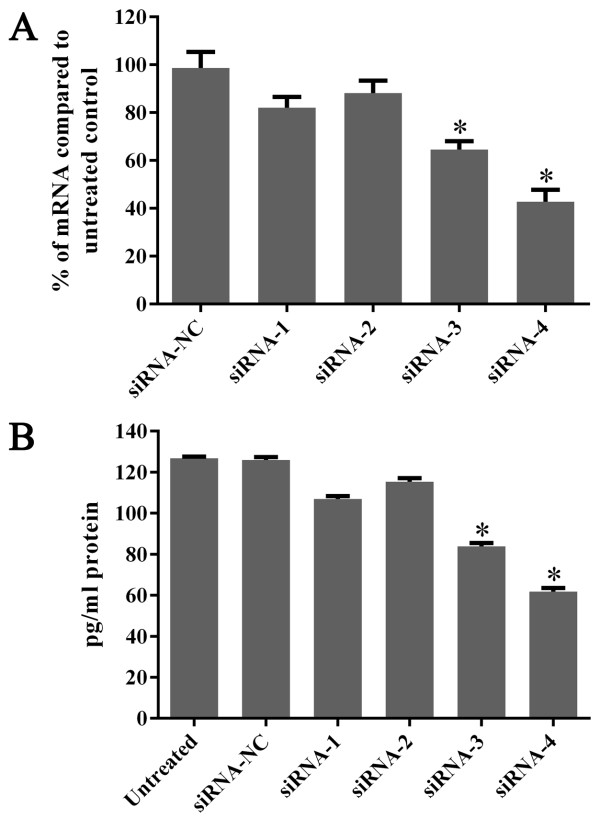
**Expression of gro-α mRNA and protein after treatment with siRNA targeted to gro-α. (A)** Detection of gro-α mRNA expression by qRT-PCR. **(B)** Detection of gro-α protein expression in cell supernatants by ELISA. ES-2 cells were treated with one of four siRNA sequences along with DharmaFECT transfection reagent for 4 h. Gro-α mRNA and gro-α protein secreted in cell supernatants were quantified 24 h or 48 h after treatment.

To determine which siRNA sequence was most effective in silencing gro-α expression, four siRNA sequences targeting gro-α mRNA were synthesized. The levels of gro-α mRNA and protein in ES-2 cells were quantified by real-time qRT-PCR and ELISA methods 24 h or 48 h after treatment with different siRNA sequences and DharmaFECT transfection reagent. As shown in Figure 2A, gro-α mRNA was down-regulated to 82.1%, 88.2%, 64.5% and 42.8% of the control level by siRNA1, siRNA2, siRNA3 and siRNA4, respectively. Consequently, gro-α protein secreted in supernatants was reduced to 84.4%, 91.0%, 66.1% and 48.7% of the control level by siRNA1, siRNA2, siRNA3 and siRNA4, respectively (Figure 2B). Thus, siRNA4 was used as the targeted sequence to suppress gro-α expression in this study.

### Characterization of FSH β 33–53 peptide-conjugated gro-α siRNA-loaded NPs

**Figure 3:**
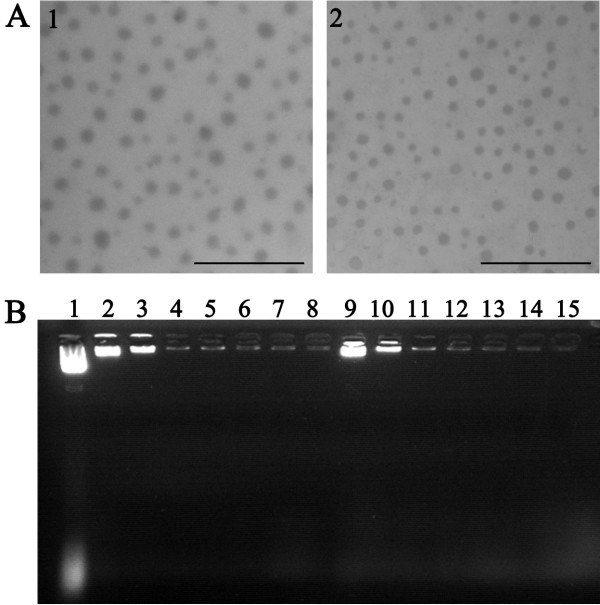
**Characterization of nanoparticle complexes. (A)** Transmission electron micrographs. 1, FSH33-G-NP; 2, G-NP. Bar, 500 nm. **(B)** The encapsulating efficiency as determined by gel retardation assay. Lane 1, naked pDNA; lanes 2 to 8, FSH33-G-NP at the N/P ratios of 1, 5, 10, 15, 20, 25 and 30, respectively; lanes 9 to 15, G-NP at the N/P ratios of 1, 5, 10, 15, 20, 25 and 30, respectively.

To prepare FSH β 33–53 peptide-conjugated gro-α siRNA-loaded NPs, gro-α siRNA4 was encoded into a linear mammalian siRNA expression plasmid, pcDNATM6.2-GW/EmGFP-miR. The complexes of gro-α siRNA-loaded NPs (G-NP) and FSH β 33–53 peptide-conjugated gro-α siRNA-loaded NPs (FSH33-G-NP) were then prepared as previously described [10]. The transmission electron micrographs of the complexes are shown in Figure 3A. Gro-α siRNA-loaded NPs modified with or without FSH β 33–53 peptide exhibited spherical shapes, with average diameters of 143.4 ± 13.2 nm and 129.2 ± 5.0 nm, respectively. The average zeta potential values were 39.8 ± 1.1 mV and 37.4 ± 2.8 mV, respectively. As shown in Figure 3B, the plasmid DNA containing gro-α siRNA was completely retarded when N/P ratios were greater than 10, which indicated an encapsulation efficiency value of 100%.

### Down-regulation of gro-α expression by FSH β 33–53 peptide-conjugated gro-α siRNA-loaded NPs

**Figure 4:**
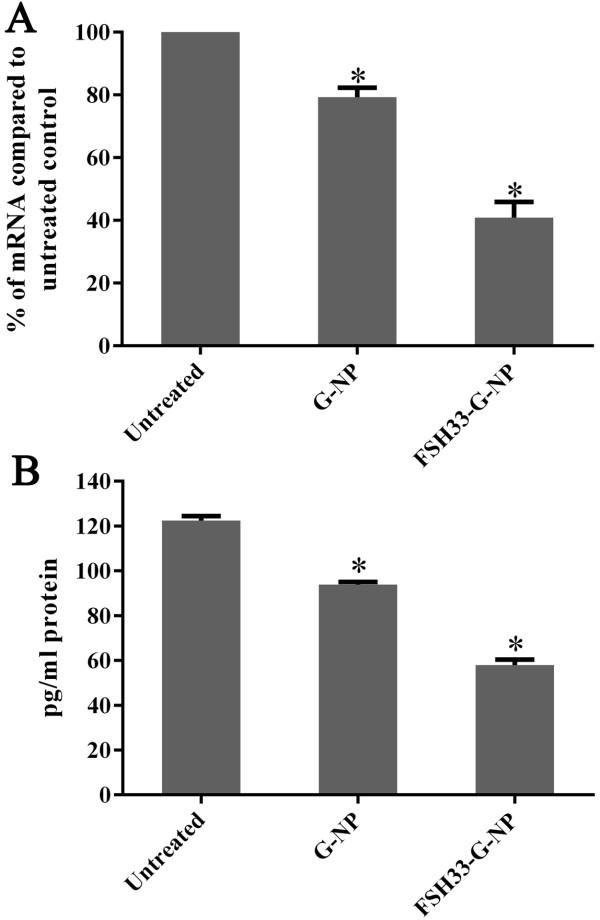
**Expression of gro-α mRNA and protein after treatment with gro-α siRNA-loaded NPs. (A)** Expression of gro-α mRNA as determined by qRT-PCR. **(B)** Expression of gro-α protein secreted in cell supernatants as determined by ELISA. ES-2 cells were treated with different nanoparticle complexes for 4 h. Gro-α mRNA and gro-α protein secreted in cell supernatants were quantified 24 h or 48 h after treatment.

We previously reported that FSH33-G-NP with an N/P ratio of 25 has a higher transfection efficiency than the complex without FSH β 33–53 peptide modification, and a higher gro-α inhibition rate (39%, down-regulated to 61% of the control level) in ES-2 cells treated with targeted complexes containing gro-α siRNA for 48 h [10]. In this study, we further investigated the suppression efficiency of gro-α expression by different complexes. Gro-α mRNA and protein levels in ES-2 cells were quantified by qRT-PCR and ELISA methods 24 h or 48 h after treatment with different complexes containing 1.5 μg of plasmid DNA. As shown in Figure 4A, gro-α mRNA was down-regulated to 79.3% and 40.9% of the control level at 24 h after treatment with G-NP and FSH33-G-NP, respectively. Consequently, levels of gro-α protein secreted in supernatants were reduced to 76.7% and 47.3% of the control level at 48 h after treatment by G-NP and FSH33-G-NP, respectively (Figure 4B). The differences between the two NP complex-treated groups were statistically significant. These data further indicated that the efficiency of suppressing the target gene gro-α could be enhanced by the targeting moiety, FSH β 33–53 peptide, which specifically recognizes FSHR expressed in ES-2 cells. Moreover, the inhibition rate of FSH33-G-NP to gro-α expression was similar to DharmaFECT transfection reagent-mediated transfection of gro-α siRNA4 (Figure 2). However, transfection reagents such as DharmaFECT and lipofectamine 2000 cannot be used *in vivo*. The complexes we synthesized could be uptaken by cancer cells without the help of transfection reagents and could be used *in vivo*.

### Suppression of aggressive biological behaviors of ES-2 cells by FSH β 33–53 peptide-conjugated gro-α siRNA-loaded NPs

**Figure 5:**
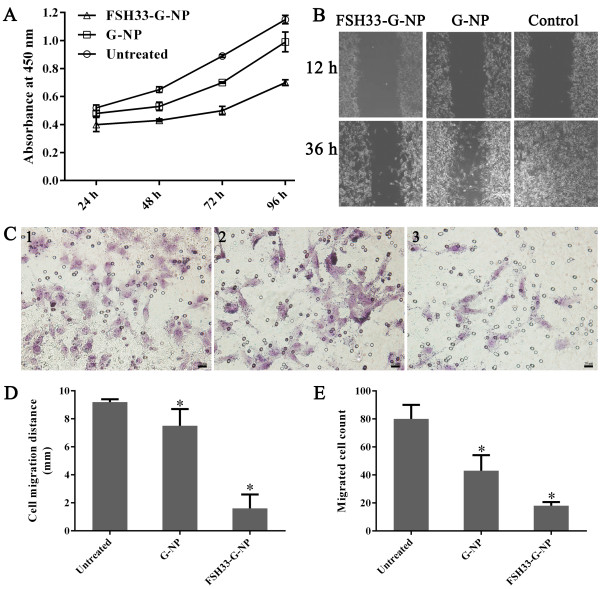
**The proliferation, migration and invasion of ES-2 cells after treatment with gro-α siRNA-loaded NPs. (A)** ES-2 cell proliferation as determined by CCK-8 assay. Cells were incubated with serum-free medium containing G-NP or FSH33-G-NP for 4 h. At 24 h, 48 h, 72 h and 96 h, CCK-8 solution was added, and OD values at 450 nm were measured. **(B)** Determination of ES-2 cell migration by scratch assay. The cell monolayer was scraped 72 h after treatment. Cells were then photographed under light microscopy at 12 h and 36 h after scraping. **(C)** Determination of ES-2 cell invasion by transwell migration assay. 1, untreated cells; 2, cells treated with G-NP; 3, cells treated with FSH33-G-NP. Bar, 20 μm. Cells were harvested at 24 h after treatment and seeded into the upper chambers of transwell plates pre-coated with matrigel. After 24 h, cells were fixed, stained and examined by light microscopy. **(D)** Determination of cell migration distances by scratch assay at 36 h. **(E)** Determination of migrated cell counts by transwell migration assay.

The proliferation, migration and invasion of ES-2 cells were investigated after ES-2 cells were treated with different complexes containing 1.5 μg of plasmid DNA containing gro-α siRNA. As shown in Figure 5A, the proliferation of ES-2 cells was inhibited by gro-α siRNA-loaded NPs modified with or without FSH β 33–53 peptide. Compared with control and G-NP, FSH33-G-NP significantly inhibited ES-2 cells. The inhibition rate at 96 h was 39.1%, which was 2.8-fold higher than that seen with G-NP. The migration ability of ES-2 cells at 36 h was also significantly inhibited by FSH33-G-NP, with a migration distance of 1.6 mm, which was 4.7-fold lower than that of cells treated with G-NP (Figure 5B and 5D). Similarly, the number of migrated cells in the FSH33-G-NP group was significantly reduced compared to that in the G-NP (without FSH β 33–53 peptide conjugation) group. The number of migrated cells in the FSH33-G-NP group was 2.4-fold lower than that in the G-NP group (Figure 5C and 5E). These data suggested that the aggressive biological behaviors of ES-2 cells were suppressed by gro-α siRNA-loaded NPs. More importantly, the targeting moiety, FSH β 33–53 peptide, significantly enhanced the suppressive effects.

## Discussion

To find an effective targeted therapy strategy for ovarian cancer and a stable delivery system for siRNA, we developed an active targeted gene delivery system mediated by FSHR and showed its promising effects for down-regulating the target gene and suppressing ovarian cancer cells.

Our group previously reported that an active targeted nanoparticle drug delivery system mediated by FSHR has a high selectivity for FSHR-positive ovarian cancer cells, and that the nanoparticles modified with FSH β 33–53 or β 81–95 peptide deliver more chemotherapeutic drugs into ovarian cancer cells and significantly enhance the antitumor efficacy of chemotherapeutic drugs [8,9,23]. Thus, FSH β 33–53 peptide was used as a targeting moiety in this gene delivery system.

However, the chemotherapeutic drugs we used before, such as paclitaxel, target ovarian cancer cells themselves rather than the whole tumor tissue. Tumors are well-organized tissues rather than a cluster of cancer cells. Tumor-associated stroma plays an important role as a niche for cancer cells and is not a passive bystander during the process of oncogenesis [24-26]. The stroma surrounding cancer epithelial cells not only provides a supportive and nutritive microenvironment for cancer cells, but it also assists in the development and progression of cancer [27-29]. The radiation-induced mammary stroma has been shown the potential to promote the neoplastic progression of non-tumorigenic mammary epithelial cells [30]. Thus, the therapeutic approaches that only aim at cancer epithelial cells could not destroy the well-organized tumor tissue thoroughly. The remaining cancer cells or premalignant epithelium could proliferate again with the support of their surrounding stromal cells including inflammatory cells, endothelial cells and fibroblasts.

In order to create a favorable microenvironment, cancer cells might have a cross-talk with their surrounding stroma. Blocking the cross-talk network could suppress the development and progression of cancer. Recent studies have reported that pro-inflammatory cytokines such as gro-α, interleukin 6 (IL-6) and interleukin 8 (IL-8) have the potential to modulate stroma and promote tumor growth [20,31,32]. A model was hypothesized by Liu to describe the inflammatory network bridging senescent stroma and tumorigenesis [33]. In this model, both initiated epithelium and senescent stroma can produce some molecules such as gro-α, IL-6 and IL-8, and then, they can promote further tumorigenesis and senescence by paracrine signaling. Gro-α and its receptor have been found to be overexpressed in ovarian cancers and play critical roles in the development and progression of ovarian cancer [20]. Therefore, siRNA to silence gro-α was introduced into the FSHR-mediated delivery system and its antitumor effects were further evaluated in this study.

High levels of gro-α are essential for malignant transformation of normal ovarian epithelial cells and can promote the proliferation of tumor cells [20,34]. It has been reported that the continuous expression of gro-α, -β, or -γ in immortalized melanocytes results in nearly 100% tumor formation in SCID mouse models. Antibodies to gro proteins are able to slow or inhibit the formation of tumors and suppress the angiogenic response [35]. Silencing of gro-α with RNAi technology also results in a 20% decrease in esophageal cancer cell proliferation [36]. In our study, the proliferation of the ovarian clear cell carcinoma cell line ES-2 was inhibited after gro-α was silenced with gro-α siRNA-loaded NPs. Compared with gro-α siRNA-loaded NPs without FSH peptide modification, gro-α siRNA-loaded NPs modified with FSH β 33–53 peptide showed an enhanced inhibitive effect to ES-2 cells. It could be due to the peptide fragments specifically binding to FSHR on ovarian cancer cells, thus greatly improving cell uptake by receptor-mediated endocytosis and internalization.

Gro-α over-expression is also involved in tumor cell migration and invasion, and ultimately promotes cancer metastasis [16,19,37]. Gro-α might be an independent predictor for bladder cancer metastasis [38]. U251 glioma cells transfected with gro-α express high levels of several proteins associated with migratory behavior and exhibit stronger motility and invasiveness. The implantation of gro-α glioma clones into the brains of nude mice form larger intracerebral tumors and cause the early death of mice [39]. In addition, CXC chemokines (including gro-α) that are secreted by endothelial cells can induce tumor cell invasion, whereas the blockade of the gro-α receptor CXCR2 can inhibit the invasion of oral squamous cell carcinoma-3 and Kaposi’s sarcoma cells into endothelial cells [40]. Similarly, the invasion and migration of breast cancer cells is markedly reduced after treatment with anti-gro antibody [41]. In colorectal cancer liver metastases, down-regulation of gro-α results in the inhibition of cell viability, invasion and proliferation in vitro and almost completely prevented tumor growth *in vivo*[42]. We examined the migration and invasion activities of ES-2 cells by cell scratch and transwell migration assays. The data showed that silencing gro-α expression markedly inhibited cell migration and invasion. Compared to gro-α siRNA-loaded NPs, the complex modified with a targeting moiety had an enhanced suppressive effect on ES-2 cells, which indicated that targeting moieties such as receptor-binding peptides could improve the intake of siRNAs carried by NPs and could enhance the effect of RNA interference.

## Conclusions

Taken together, this study indicated that silencing gro-α expression suppressed the proliferation, migration and invasion of ovarian clear cell carcinoma cells and that the FSHR-mediated nanoparticle delivery system provided a highly efficient delivery tool for gro-α siRNA into FSHR-expressing cells. Thus, silencing gro-α by a receptor-mediated targeted strategy is a potential choice for ovarian cancer treatment. However, further studies *in vivo* are required to investigate the therapeutic effects of this targeted complex in ovarian cancer.

## Abbreviations

siRNA: Short interfering RNA; RNAi: RNA interference technology; NPs: Nanoparticles; FSH: Follicle-stimulating hormone; FSHR: Follicle-stimulating hormone receptor; PEG: Polyethylene glycol; PEI: Polyethylenimine; gro-α: Growth regulated oncogene α; Mal: Maleimide; CCK-8: Cell counting kit-8; ELISA: Enzyme-linked immunosorbent assay; RT-PCR: Reverse transcription-polymerase chain reaction; pDNA: Plasmid DNA; qRT-PCR: Real-time quantitative RT-PCR; OD: Optical density; G-NP: Gro-α siRNA-loaded NPs; FSH33-G-NP: FSH β 33–53 peptide-conjugated gro-α siRNA-loaded NPs; IL-6: Interleukin 6; IL-8: Interleukin 8.

## Competing interest

We declare that we have no conflict of interest.

## Authors’ contributions

SSH performed the experiments and drafted the manuscript. JC and YFZ participated in the design of this study and edited the manuscript. JBZ participated in the experiments. XYZ and CJX contributed to the design of this study, final data analysis and edited the manuscript. All authors read and approved the final manuscript.
